# Analgesic effects of lidocaine-ketorolac compared to lidocaine alone for intravenous regional anesthesia

**DOI:** 10.22088/cjim.9.1.32

**Published:** 2018

**Authors:** Shahram Seyfi, Nadia Banihashem, Ali Bijani, Karimollah Hajian-Taliki, Mohsen Daghmehchi

**Affiliations:** 1 Cancer Research Center, Health Research Institute, Babol University of Medical Sciences, Babol, Iran; 2 Department of Anesthesiology, Clinical Research Development Unit, Ayatollah Rouhani Hospital, Babol University of Medical Sciences, Babol, Iran; 3 Non-Communicable Pediatric Diseases Research Center, Health Research Institute, Babol University of Medical Sciences, Babol, Iran; 4 Social determinat Health ResearchCenter, Health Research Institute, Babol University of Medical Sciences, Babol, Iran; 5 Student Research Committee, Babol University of Medical Sciences, Babol, Iran

**Keywords:** Lidocaine, ketorolac, pain, Intravenous regional anesthesia

## Abstract

**Background:**

Intravenous regional anesthesia is a simple and reliable method for upper extremity surgery. In order to increase the quality of blocks and reduce the amount of pain, many drugs are used with lidocaine. In this study, the effect of ketorolac-lidocaine in intravenous regional anesthesia was investigated.

**Methods:**

40 patients undergoing elective upper limb with America Society of Anesthesiologists class I and II were selected and randomly divided into two groups. The first group of 20 patients received 200 mg of lidocaine, and the second group, 200 mg of lidocaine with 20 mg of ketorolac. In both groups, the drug was diluted to 40 ml. In both groups, the onset of sensory block, onset of tourniquet pain, the onset of pain after opening the tourniquet, score of postoperative pain and analgesic prescription in the first 24 hours, during 1, 6, 12 and 24 hours were studied. A measure of the quality of analgesia was evaluated by VAS.

**Results:**

The mean onset of tourniquet pain in the two groups was not significantly different (P=0.443). In the ketorolac group, the onset of pain after opening the tourniquet was significantly longer than lidocaine group (p<0.001). The mean postoperative pain score during the first 24 hours after surgery in the ketorolac group was significantly lower than lidocaine group (p<0.001). The average number of analgesia prescription during the 24 hours after operation was significantly lower in ketorolac group than lidocaine group (p<0.001).

**Conclusions:**

Adding ketorolac to lidocaine for regional anesthesia can reduce the postoperative pain for up to 24 hours after opening the tourniquet.

Intravenous regional anesthesia is a simple but reliable method for upper extremity surgery and sometimes used for lower limb. This method is excellent and acceptable because of the good muscle relaxation, rapid onset, fast recovery and its success in the surgeries less than 90 minutes(-). In this procedure, a small catheter is inserted into a vein in the end part of specified limb (4). In this way, blood drain was done using a plastic Esmarch bandage with easy and flexible comfort (-). The most commonly used anesthetic drugs include lidocaine and prilocaine (4, 5). Lidocaine is widely used and acceptable drug by the Food and Drug Association of USA (FDA). Other drugs such as prilocaine, procaine, chloroprocaine, bupivacaine or rpivacaine are used less or set aside or need further studies (6). Many drugs were used with lidocaine to increase the quality of block and reduce the amount of pain, including opioids, dexmethomedine, magnesium sulfate, sodium bicarbonate ketamine, nonsteroidal anti-inflammatory drugs, etc (6). Many drugs and methodologies were used to relieve postoperative pain, including opioids, ketorolac (-) and N acetyl cysteine on pain after laparoscopic cholecystectomy (9), Foot and hand massage on post- cesarean section pain (10). 

Ketorolac, which has anti-inflammatory and pain relieving properties, is the only nonsteroidal anti-inflammatory drug confirmed to be safe to administer intravenously. Its complications are the same as the other drugs in this group, with ketorolac only with milder gastrointestinal complications. The drug can be used for a maximum of 5 days. It decreases the pain and increases the period of painlessness. Therefore, it is added to lidocaine in venous blocking for postoperative pain relief (4). The advantages of this method include: lack of intubation complications, nausea and vomiting after surgery, easy prescription, fast recovery, rapid onset and adequate muscle relaxation (4, 5). 

Severe complications of intravenous regional anesthesia are rare and it does not have any complications like those related to epidural and spinal and general anesthesia  (7, 11). The other side effects of this method include bruising, infection, blood clots and thrombosis, arterial spasm, allergic reactions and skin discoloration  (7, 11). The side effects of this method with local anesthetic poisoning include symptoms of dizziness, nystagmus, drowsiness and decreased blood pressure and heart rate, cardiac output, and sometimes cardiovascular collapse (4, 5).

Bier block has replaced general anesthesia in many hand surgeries.In recent studies, this approach has been used in the treatment of excessive palm sweating and in reducing pain of botulinum toxin injections into the palm (100 ml in each hand) (12, 13). After releasing the touniquet, the effect of lidocaine wears off quickly. The patient then starts to feel pain and narcotic or non-narcotic painkiller- may be needed which has its own complications. Therefore, ketorolac is added to increase postoperative painlessness and reduce the use of narcotics (4). This study aimed to investigate the analgesic effect of lidocaine with ketorolac in the intravenous regional anesthesia method.

## Methods

This double- blind prospective, randomized clinical trial was performed at Shahid Beheshti Hospital during May to December 2015. This research project was approved by the thics Committee of Babol University of Medical Sciences. The study was registered in the Iranian registration system (IRCT2015082420020N2). 40 patients with inclusion criteria such as: age between 20-70 years; candidate for elective surgery wrist and forearm fractures; Grade 2 1 The America Society of Anesthesiologists (healthy patients without the disease) were selected and divided randomly into two groups. Surgical treatments for hand, wrist and forearm fractures were the same. Exclusion criteria were as follows: patients aged over 70 years and previous emergency hand surgery; known hypersensitivity to ketorolac; surgery more than 1 hour; an unusual complication of surgery and anesthesia intubation; chronic pain in the hands before surgery; mental illness and psychological disorders; drug use; pregnancy; breastfeeding and other simultaneous surgery.

Patients initially underwent routine monitoring, such as pulse oximetry, ECG, NIBP. For all patients, the same premedication was prescribed which included 200 ml normal saline, 1.5 mg of midazolam and 75 mg fentanyl. Then, after insertion of intravenous catheter in the organ under surgery, full drying of arm vein was done using Esmarch band and tourniquet.

After ensuring tourniquet inflation and lack of pulse in the control group of 20 patients, 200 mg of lidocaine (lidocaine group) was injected; while in the intervention group of 20 patients, 200 mg of lidocaine and 20 mg of ketorolac (ketorolac group) was injected in the hand during surgery. Since the study was double-blind and to prevent any error, the liquid volume of all syringes in both control and intervention groups was at an equal volume of 40 ml and was specified with numbers 1 and 2, that only a collaborator was aware of these numbers. The assistant project manager was not aware of drug injection.

In both groups, the onset of sensory block, analgesia quality, onset of tourniquet pain during operation and pain in intra-operative anesthesia and tourniquet were measured. Sensory block was evaluated every minute and lack of feeling of a sharp needle in all relevant dermatomes mentioned by patient was recorded as the time of onset of sensory block. Analgesia quality was measured based on VAS (zero: no pain, 10: worst pain experienced). After the operation, the tourniquet (at least 30 minutes after administration of the drug) was opened slowly over a time period of about 30 seconds. 

All surgeries lasted less than an hour. The onset of pain after opening the tourniquet and the quality of analgesia in recovery was assessed during the 24 hours. Patients with pain scored more than six in the recovery received 20 mg of pethidine and 1 g Apotel, if necessary, at any time within 24 hours of hospitalization and the mean frequency of analgesic prescribed (pethidine and Apotel) was evaluated in two groups. The data were analyzed by t-test, Anova, repeated measures, Fisher’s exact test and regression model where possible. A p<0.05 was considered as significant.

## Results

Among the total cases under study, 30 (75%) patients were males and 10 (25%) patients were females. The average age of patients in the ketorolac-lidocaine group was 34.05±12.64 years and 32.65±11.92 years in the lidocaine group, the mean age had no significant difference between two groups (P=0.711). The mean onset of sensory block in the lidocaine group was 3.96±1.02 minutes, and in the ketorolac group was 4±0.91 minutes; the two groups showed no significant difference in this regard (P=0.855). The mean onset and tourniquet pain score were not significantly different between the two groups. 

The onset time of pain in the hand under surgery after opening tourniquet was higher in the lidocaine group than the ketorolac group which was significantly different. The mean pain score in recovery for lidocaine group was higher than the ketorolac group; there was a significant difference between the two groups (table 1).

**Table 1:** The mean onset and score of tourniquet pain and pain after the opening tourniquet in both intervention and control groups

**P value**	**Ketorolac-Lidocaine** **mean±SD**	**Lidocaine alone** **Mean±SD**	**Variables**
0.996	34.25±7.99	34.26±4.96	Onset of tourniquet pain (min)
0.768	2.5±3.23	2.78±2.99	Score of tourniquet pain VAS (0-10)
0.001	264±70.59	12.13±3.78	The duration of analgesia after opening tourniquet (min)
0.001	1.9±0.44	6.26±1.38	Score of pain in the recovery VAS (0-10)

**Fig 1:**
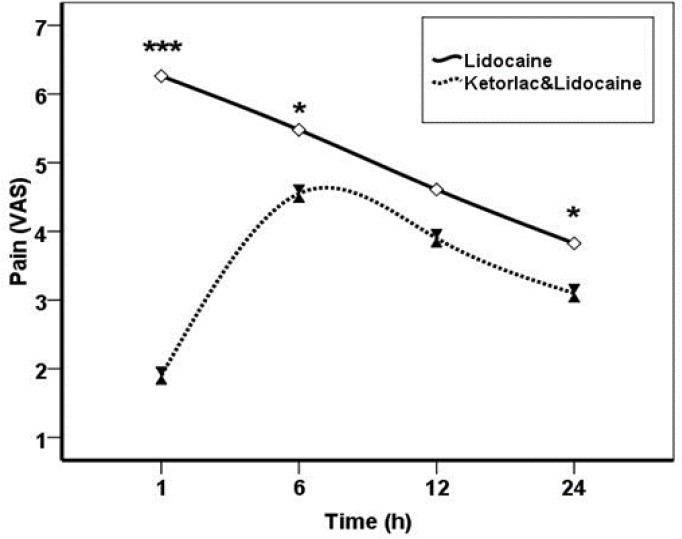
The average pain score during different intervals of 24 hours in two groups

The mean pain score at 1, 6 and 24 hours for the lidocaine group was higher than the ketorolac group; there was a significant difference between the two groups. But 12 hours after surgery, this difference was not significant (table 3) In general, postoperative pain score during the first 24 hours after surgery in the ketorolac group was always lower than lidocaine group (P=0.048) (fig 1) 5 patients of the lidocaine group only received pethidine during recovery, but none of the patients of ketorolac group received pethidine during recovery. There was a significant difference between the two groups (P<0.001). The average doses of Apotel administered in ketorolac group was 2.09±0.90, and 0.8±1.15 in lidocaine group, which was significantly different between the two groups (P<0.001).

**Table 3:** The average pain score [VAS (0-10)] in both groups during 24 hours

**Pvalue**	**ketorolac-lidocaine ** **mean±SD**	**Lidocaine alone** **Mean±SD**	**Variables** **Pain Score**
0.001	1.9±0.44	6.26±1.38	1 hour
0.032	4.55±1.63	5.48±1.08	6 hours
0.069	3.9±1.51	4.61±0.94	12 hours
0.048	3.10±1.37	3.83±0.83	24 hours

## Discussion

The mean time of tourniquet pain and onset of sensory block were similar in both groups. The onset of pain after opening tourniquet, the mean pain score and the mean number of analgesic doses administered during the first 24 hours after surgery in ketorolac group was significantly lower than lidocaine group. In this study, onset of sensory block in the lidocaine group was slightly less than ketorolac group, but there was no significant difference, which was in line with other studies; this result suggests that ketorolac had no important influence on the onset of anesthesia. 

Singh-R in a comparison study between two intravenous regional anesthesia methods with lidocaine and ketorolac on forearm and upper arm in two groups of 20 samples, found that the onset of sensory block did not differ between two groups; but duration of analgesia onset in the ketorolac group was slightly higher (14). Other studies also indicated that adding ketorolac at the time of onset of anesthesia had no effect (-). 

In our study, the mean onset and score of tourniquet pain in lidocaine group was higher than ketorolac group. There was no significant difference between the two groups. In the study of MyoungJinKo comparing the effect of ketorolac and paracetamol and lidocaine in intravenous regional anesthesia, the onset of tourniquet pain was not significantly different in the three groups (18). In few studies, the intravenous regional anesthesia by the use of ketorolac with other drugs had no significant difference in terms of tourniquet pain (19, 20). In a study of Reuben SS on the application of lidocaine and ketorolac in intravenous regional anesthesia on 60 cases divided them into two groups of lidocaine-ketorolac and lidocaine alone, patients who received ketorolac experienced significantly decreased tourniquet pain during the operation, which is incompatible with our study (21).

In the study of Abdel-Ghaffar, in which ketamine was added to lidocaine, indicated that tourniquet pain in group ketamine group was less than the lidocaine group (22) that is due to the analgesic effect of ketamine.Our study showed that the 20 mg ketorolac in regional intravenous bolus has proper analgesis between 4 to 6 hours (264±70.59) after surgery. In the study of Robert B. Steinberg on the effective dose of ketorolac for intravenous regional anesthesia, pain level in groups of 20 and more than 20 mg ketorolac was significantly less than those groups that received less than 20 mg of ketorolac (23). Many other studies confirmed the effect of intravenous ketorolac in delaying the onset of pain after surgery (17, 20, 21, -).

The existing study showed that ketorolac group had significantly prolonged the duration of analgesic use after surgery and pain score in recovery (1.9±0.44) and it was significantly lower than the control group during 24 hours in 1, 6, 24 hours after surgery. Ketorolac with systemic absorption after opening tourniquet can control the cyclo-oxygenase enzyme and reduce pain. In a study conducted by Reuben SS on the application of lidocaine and ketorolac in intravenous regional anesthesia, pain score during the first 24 hours after surgery in lidocaine-ketorolac group was significantly less than lidocaine alone (p<0.05) (21). In other studies, similar results were obtained for intravenous regional anesthesia procedure (16, 17, 23, 25, 28, 29). The current study also showed that ketorolac can significantly reduce the consumption of analgesic drugs and Apotel during the first 24 hours after the operation. Reduction of painkillers based on pain score and the reason of less pain in the ketorolac group was the analgesic effect of ketorolac. Myoung Jin Ko conducted a study about the effect of ketorolac and paracetamol on the intravenous regional anesthesia in patients, which were divided into three groups of lidocaine alone, lidocaine-paracetamol and lidocaine-ketorolac. Average pain and the need of painkillers (tramadol) in the group with ketorolac were less than the other two groups (18). In multiple studies, the requirement of additional drug in the ketorolac group was significantly lower than lidocaine group (23, 26, -). In our study, only about 25 percent of patients in the lidocaine group needed pethidine after surgery and in recovery. In the study of Arregui-Martínez de Leganza LM, 23% of lidocaine group consumed painkillers, while no patients in the ketorolac group needed painkillers, which had statistically significant differences (p<0.0001) (32). With regard to the limitations of this study, ensuring the accuracy of the answers was beyond the scope of this research, in addition to long time follow-up of patients, failure to control the use of non-narcotic analgesics, unwillingness of the patient and allergy and anaphylactic reaction.

In conclusion, it was found that the postoperative pain score during the first 24 hours after surgery in the ketorolac group was always lower than the lidocaine group. According to the results of the study, it was indicated that ketorolac group experienced better quality of analgesia than the lidocaine group and the amount and duration of pain after opening tourniquet was lower.
